# Anticoagulant-related bleeding in patients receiving anticoagulant therapy over 10 years

**DOI:** 10.1186/s13054-023-04646-9

**Published:** 2023-09-18

**Authors:** Lihong Zhu, Juan Lin

**Affiliations:** Intensive Care Unit, Zhejiang Hospital, 12# Linyin Road, Hangzhou City, 310013 Zhejiang Province China

To the Editor,

In a recent study, Dr. Botrel et al. [1] summarized the characteristics of patients admitted to intensive care units (ICU) for severe anticoagulant-related extracranial bleeding (AREB) in five centers. This study is well designed. However, several limitations should be noted.

First, a total of 95,614 patients admitted to five French ICUs from January 2007 to December 2018 were initially screened. The authors reported that the incidence of AREB increased from 3.2/1000 (18 patients) in 2007 to 5.8/1000 (56 patients) in 2018, which requires medical attention. This result may be incorrectly interpreted.

In this study, the authors used the entire ICU population (*n* = 95,614) as the denominator in AREB incidence calculations, which could lead to a biased result. According to the data provided, the total ICU admissions increased from 5625 (18*1000/3.2) in 2007 to 9655 (56*1000/5.8) in 2018. However, over the past decade (2007 to 2018), the proportion of patients receiving anticoagulation therapy has also gradually increased. For instance, in China, the percentage of atrial fibrillation patients receiving anticoagulation therapy increased from 2.7% in 2002 [2] to 31.7% in 2012 [3]. Therefore, it is more reasonable to use the number of people receiving anticoagulation therapy as the denominator when calculating the AREB incidence. When the proportion of patients receiving anticoagulant therapy increases significantly over 10 years, the overall AREB incidence may decrease (for instance, assuming the proportion of patients receiving anticoagulation therapy increased from 15% in 2007 to 60% in 2018 in France, the AREB incidence would decrease from 2.13% (18/(5625*15%)) to 0.96% (56/(9655*60%)). This is also consistent with the fact that over time, more people received oral anticoagulants, and dozens of trials [4] have reported that oral anticoagulants have a lower risk of bleeding than traditional anticoagulants such as heparin. Therefore, in theory, the overall probability of bleeding events should not increase.

Second, in the multivariate logistic model, the author kept all variables (*n* = 17, including non-significant factors) in the final model and found that only five risk factors remained significant. This may not be appropriate. A logistic regression model [5] could be over-parameterized (i.e., too many variables for too few events) and can result in odds ratio uninterpretable (i.e., 95% CI extremely large or reversed). This may be why some known risk factors (such as age, MV, and RRT) for ICU mortality became non-significant in this model. In addition, the disease severity score (SOFA or SAPS II) should be adjusted to reach a stable result.

## Data Availability

Not applicable.
